# Supplementary material for: SNPs Sets in Codifying Genes for Xenobiotics-Processing Enzymes Are Associated with COPD Secondary to Biomass-Burning Smoke
Source: Curr Issues Mol Biol. 2023 Jan 17;45(2):799–819. doi: 10.3390/cimb45020053 (PMC9954820; doi:10.3390/cimb45020053)
Supplement: Supplementary file 1 [file cimb-45-00053-s001.zip › cimb-2119150-supplementary.pdf]

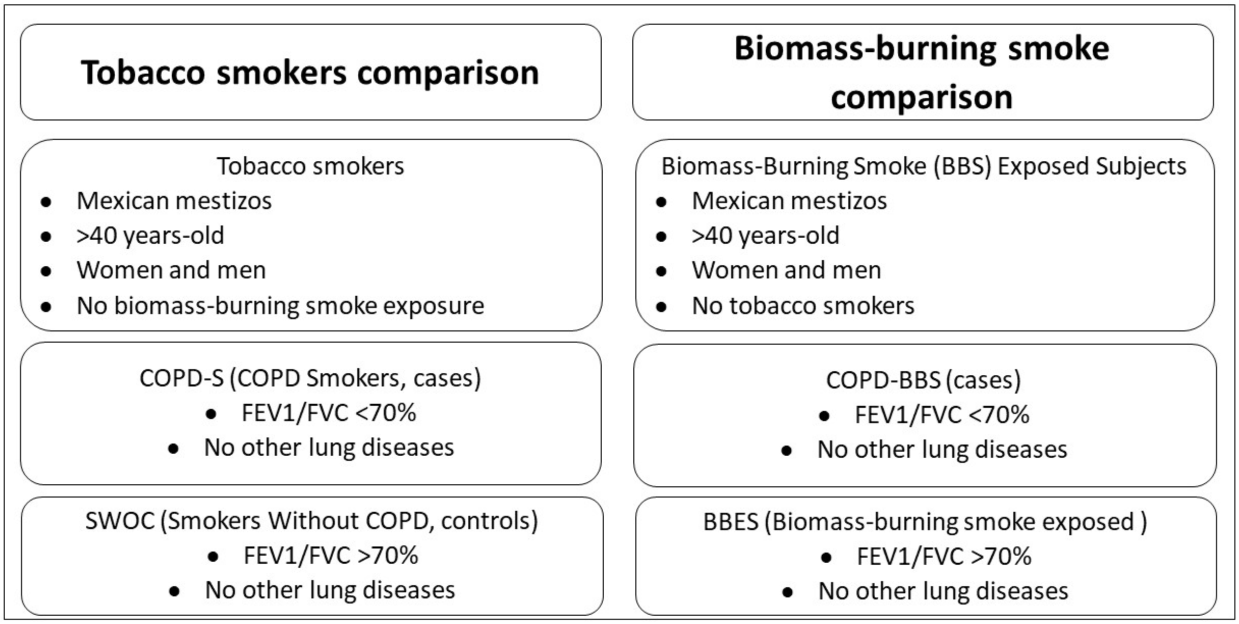

Supplementary Figure S1. Sample selection criteria for both comparison groups. FEV1: Forced expiratory volume at 1st second, FVC: Forced vital capacity.

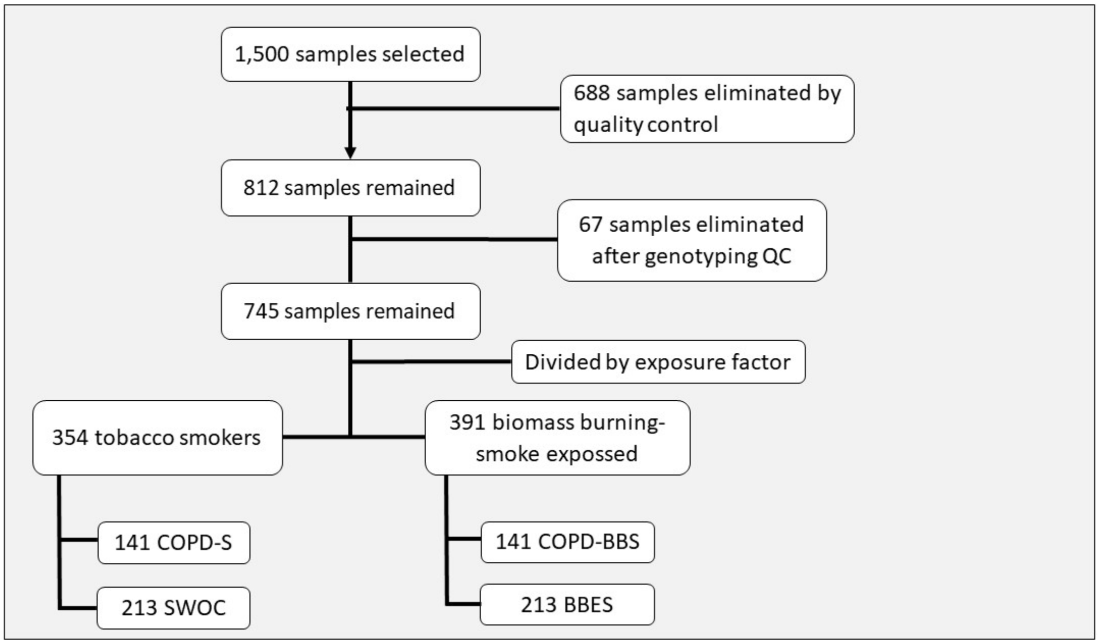

Supplementary Figure S2. Sample selection flowchart for both comparison groups. QC: quality control.

Supplementary Table S1. List of SNPs included in the analysis.

CHR	SNP	CHR	SNP	CHR	SNP	CHR	SNP
1	rs2297813	1	kgp15775548	10	exm-rs1004467	15	variant.35738
1	exm57055	1	exm95198	10	exm852673	15	exm1161553
1	exm57057	1	rs2275237	10	rs3781287	15	exm1161560
1	rs837398	1	exm1713582	10	kgp2582226	15	exm1161562

1	rs681840	1	exm95207	10	rs762563	15	rs6493489
1	rs1105456	1	exm95209	10	exm852682	15	exm1161578
1	rs1890250	1	exm95215	10	kgp21904172	15	variant.35739
1	exm57064	1	kgp9229586	10	rs743572	15	exm1833786
1	exm57067	1	rs2229175	10	kgp29884787	15	exm-rs2899472
1	exm57068	1	rs17855056	10	exm854593	15	rs2414095
1	exm57069	1	exm95240	10	exm854612	15	variant.35741
1	rs2297812	1	exm95244	10	rs4925	15	rs700518
1	kgp15329500	1	kgp2698943	10	exm854616	15	exm-rs16964211
1	kgp24935388	1	exm95253	10	kgp5950177	15	exm2223505
1	exm57078	1	rs10305679	10	exm854617	15	exm1161608
1	exm57079	1	rs17855059	10	variant.14531	15	rs16964220
1	rs4646487	1	exm95278	10	exm1670407	15	rs1008805
1	exm57093	1	rs4147592	10	rs17116751	15	rs749292
1	exm57103	1	rs4147594	10	exm854619	15	exm-rs2305707
1	exm2233505	1	rs4147595	10	kgp2910370	15	rs8029807
1	exm57109	1	newrs9333378	10	rs15032	15	rs936306
1	exm57110	1	rs10800120	10	exm854622	15	rs10519301
1	exm57112	1	rs9333413	10	kgp21589222	15	rs10519302
1	kgp15405951	1	rs957644	10	kgp7613533	15	rs2470144
1	exm57113	1	exm119609	10	kgp21587903	15	variant.36725
1	exm57115	1	exm119611	10	kgp21623870	15	exm1176450
1	exm57117	1	var_1_165620270	10	kgp21779935	15	rs6161
1	SNP137	1	rs11799886	10	kgp21895818	15	kgp3520678
1	exm57125	1	exm119620	10	exm854629	15	exm1176461
1	exm2233506	1	exm119621	10	exm854635	15	rs6160
1	exm57127	1	exm119633	10	var_10_106037836	15	exm1176465
1	exm57128	1	rs8133	10	exm1670419	15	exm1176466
1	exm57129	1	rs4147611	10	exm854649	15	exm1176471
1	rs2297810	10	exm842287	10	rs156697	15	kgp28340310
1	exm57130	10	exm842298	10	exm854650	15	rs11632698
1	exm57131	10	exm2249435	10	rs276203	15	rs1484215
1	exm57133	10	exm842301	10	rs10437455	15	kgp19805840
1	exm57137	10	exm842308	10	rs156699	15	kgp3956993
1	exm57139	10	exm842309	10	exm854654	15	exm1176481
1	kgp1241099	10	exm842311	10	exm854657	15	exm1176488
1	exm57140	10	exm842319	10	exm854663	15	rs1130841
1	exm57152	10	exm842320	10	kgp22053300	15	rs11544450
1	exm57155	10	exm842322	10	variant.14541	15	kgp19913951
1	exm57157	10	exm842335	10	exm857160	15	kgp19964059
1	exm57160	10	exm842346	10	exm857173	15	kgp19794882
1	exm57161	10	exm2217665	10	exm857174	15	kgp28430319
1	exm57163	10	kgp5493839	10	exm857186	15	kgp19982895

1	kgp6364747	10	exm842392	10	exm857199	15	kgp19967485
1	kgp8583761	10	exm2217666	10	exm857200	15	kgp19915208
1	exm2264832	10	exm1687884	10	kgp5418137	15	exm2223805
1	rs10749865	10	exm842399	10	exm1675891	15	exm1176854
1	rs6679068	10	exm842404	10	kgp29806682	15	exm1176857
1	rs9333035	10	exm842420	10	rs915906	15	exm1176861
1	rs9333016	10	rs35768328	10	newrs74771533	15	rs1048943
1	exm57193	10	kgp22038226	10	exm868732	15	exm1176862
1	kgp15545556	10	rs2229104	10	exm868742	15	exm1839877
1	var_1_47400671	10	kgp12115920	10	exm868746	15	exm1176867
1	exm57233	10	kgp29750936	10	exm868747	15	exm1176870
1	exm2233507	10	kgp8085304	10	exm1675899	15	variant.36755
1	exm57250	10	variant.13832	10	kgp21896191	15	rs2856833
1	rs9333005	10	exm843816	10	exm2216933	15	exm1839883
1	rs9333003	10	kgp21685072	10	kgp1074564	15	SNP31
1	exm57266	10	rs2901783	10	rs743534	15	variant.36757
1	exm57267	10	exm843828	10	kgp29696689	15	variant.36758
1	rs9332998	10	exm843830	10	exm868780	15	exm1176880
1	rs2036462	10	rs2296681	10	exm868786	15	exm1176883
1	exm57293	10	exm2249444	10	SNP127	15	SNP76
1	variant.3312	10	exm843840	10	exm868787	15	variant.36759
1	rs9793989	10	exm843843	10	exm868789	15	variant.36760
1	var_1_47497152	10	exm843844	10	rs2515641	15	exm1176890
1	exm57298	10	exm843849	10	exm868796	15	exm1176891
1	exm57301	10	kgp21803429	10	kgp21574411	15	exm1176895
1	exm57313	10	kgp4036188	10	kgp5910402	15	exm1176898
1	exm57317	10	exm843851	10	kgp21973149	15	exm1176905
1	rs11211422	10	kgp21992538	10	kgp21790543	15	exm1176910
1	exm57330	10	exm843860	11	rs7123257	15	exm1176911
1	exm57337	10	kgp10903269	11	rs7107287	15	exm1176921
1	variant.3318	10	kgp21815302	11	rs7949336	15	exm1176923
1	exm57344	10	kgp29967819	11	rs10741616	15	exm1176924
1	exm57350	10	variant.13841	11	rs7126796	15	exm1176926
1	exm57351	10	exm843871	11	exm2267150	15	exm1176927
1	rs11211436	10	exm843876	11	rs1982350	15	exm1176935
1	exm57372	10	kgp4370764	11	rs7924734	15	exm1176941
1	exm57376	10	rs3740367	11	rs10832027	15	exm1176944
1	exm2233517	10	rs1042192	11	rs16912751	15	exm1176946
1	rs6690005	10	kgp29703069	11	rs7937060	15	exm1176949
1	newrs149156333	10	kgp9175734	11	exm891861	15	exm2223809
1	kgp15820763	10	rs1042194	11	exm891911	15	exm1176951
1	rs34734216	10	kgp5376533	11	rs969485	15	exm1176954
1	exm57408	10	kgp21613472	11	exm892409	15	exm1176957

1	exm57409	10	SNP69	11	exm892419	15	exm1176958
1	exm57417	10	rs7916649	11	exm1702325	15	exm1176960
1	exm57421	10	exm843895	11	kgp5752318	15	exm1176967
1	exm57427	10	rs28399505	11	exm892421	15	exm1176975
1	rs2056898	10	exm843896	11	kgp12867434	15	exm1176983
1	exm57446	10	kgp7668749	11	exm892437	15	exm1176986
1	rs10789501	10	variant.13845	11	variant.16864	15	rs17861155
1	exm57448	10	variant.13846	11	exm892446	15	exm1839908
1	newrs112811686	10	kgp29673168	11	rs1993116	15	exm1176991
1	exm1820433	10	exm843903	11	rs7129781	15	exm1176994
1	kgp12171305	10	exm843922	11	rs12794714	15	exm1176995
1	kgp5898133	10	rs4388808	11	exm892456	15	rs28399418
1	kgp15696966	10	rs28399510	11	exm892462	15	exm1177000
1	exm64021	10	exm843929	11	exm932320	15	exm-rs2472304
1	exm2252415	10	newrs4986893	11	exm1731561	15	exm1177002
1	rs1056596	10	exm843930	11	exm932333	15	exm2274770
1	exm64040	10	rs4244285	11	variant.19580	15	rs2960193
1	exm64041	10	newrs72558186	11	exm2219297	15	newrs56107638
1	exm64044	10	exm-rs12767583	11	exm932361	15	exm1177015
1	exm64046	10	exm2267120	11	rs8191444	15	exm1177016
1	variant.3851	10	rs1853205	11	exm933748	15	exm1177019
1	exm64048	10	kgp21995418	11	exm933755	15	exm1177033
1	newrs150250306	10	variant.13853	11	exm933757	15	exm2223813
1	rs11572279	10	rs10786172	11	exm933762	15	kgp4058862
1	exm1845358	10	rs28399513	11	exm933764	15	rs12591286
1	exm64055	10	kgp21942249	11	rs1138272	15	rs8041826
1	newrs143860781	10	rs11592737	11	exm933772	15	rs7172914
1	exm64060	10	exm843958	11	rs4986949	15	rs3848208
1	SNP47	10	SNP50	11	exm933774	15	rs11635014
1	SNP101	10	exm843975	11	kgp10309627	15	rs4238518
1	exm64070	10	exm843980	11	GA028194	15	rs1037124
1	exm64074	10	rs12414460	12	rs1913263	15	rs1374213
1	exm64075	10	exm844029	12	exm987854	15	rs3901896
1	exm2233649	10	kgp21755054	12	rs4149192	15	rs7168908
1	kgp15417871	10	rs9332130	12	variant.23476	15	exm2223916
1	exm1845370	10	rs9332168	12	exm987857	15	rs2278709
1	exm64080	10	exm844034	12	rs4149197	15	rs8028295
1	kgp15360395	10	rs1856908	12	rs7312090	15	rs4778795
1	exm1845377	10	exm-rs10509680	12	rs9332944	15	rs7183286
1	rs11572191	10	kgp22009765	12	rs11875	15	rs4778799
1	exm64090	10	exm844046	12	kgp18860019	15	rs7178902
1	kgp3155172	10	SNP66	12	rs9332945	15	rs4331301
1	kgp25277353	10	kgp21943453	12	rs4149206	15	exm1182031

1	rs3191485	10	exm844050	12	rs2160512	15	rs11856676
1	exm81780	10	exm2249447	12	rs9332959	15	rs4238522
1	rs3211196	10	exm844054	12	rs6488842	15	rs12148133
1	exm81787	10	variant.13860	12	rs11048977	15	exm1182038
1	kgp7859532	10	rs9332220	12	rs1037924	15	exm1182047
1	kgp15428935	10	rs17847032	12	rs17497857	15	exm1182049
1	exm81798	10	exm1688514	12	rs4964053	15	rs4074666
1	kgp15844410	10	rs28371687	12	rs7138982	15	exm1182063
1	rs625456	10	rs2017319	12	exm991192	15	exm2274779
1	exm1692076	10	exm844070	12	exm991195	15	var_15_80855585
1	variant.5472	10	kgp537287	12	exm991196	15	rs11635554
1	exm81836	10	variant.13861	12	rs6487604	15	exm1182070
1	variant.5475	10	exm844073	12	rs4964059	15	exm1182076
1	exm81844	10	kgp22034059	12	variant.23838	15	variant.37065
1	exm81845	10	kgp21913328	12	rs12300289	15	rs7177543
1	rs655315	10	rs9332244	12	exm991217	15	rs4301984
1	rs12068997	10	kgp29854121	12	exm991219	15	rs6495511
1	exm2230743	10	rs1058932	12	exm991220	15	newrs76861207
1	exm81898	10	exm844087	12	rs1562048	15	rs1139651
1	var_1_110255320	10	rs11572177	12	newrs138112721		
1	exm81964	10	rs1934953	12	exm991224		
1	exm81966	10	exm-rs1934951	12	exm991225		
1	kgp25041089	10	exm844091	12	exm991229		
1	exm81979	10	exm844093	12	rs1037920		
1	kgp10114047	10	kgp22047705	12	exm991241		
1	exm81994	10	exm844097	12	exm991251		
1	exm81997	10	exm844098	12	exm991253		
1	kgp15708162	10	exm844102	12	rs776195		
1	kgp15419817	10	variant.13863	12	rs12297276		
1	kgp24902563	10	exm844109	12	exm1017678		
1	rs11807	10	rs11188150	12	variant.25699		
1	kgp15817546	10	exm844115	12	exm1017687		
1	kgp15192508	10	exm844119	12	exm1017697		
1	kgp5736156	10	exm844121	12	exm2251099		
1	kgp15450425	10	exm2217697	12	exm1017713		
1	rs12087868	10	rs11572103	12	exm1017716		
1	kgp5336935	10	exm844133	12	exm1017730		
1	rs12058634	10	exm844138	14	rs7303		
1	rs12040968	10	rs11572101	14	exm1116014		
1	kgp24961028	10	rs11572093	14	exm1117541		
1	kgp15859527	10	variant.13867	14	exm1117547		
1	kgp10666312	10	newrs146962089	14	exm1117549		
1	kgp15371388	10	exm844148	14	exm1117553		

1	rs2234696	10	exm844149	14	exm1117556		
1	exm81999	10	exm844150	14	newrs148051919		
1	variant.5483	10	exm844152	14	exm1117569		
1	newrs149969876	10	rs3752988	14	exm1117575		
1	rs1803687	10	variant.13871	14	exm1117582		
1	variant.5486	10	exm844157	14	exm1117583		
1	exm1692455	10	rs11572076	14	exm1820986		
1	exm82022	10	variant.13874	14	exm1117584		
1	exm82029	10	variant.13876	14	exm1117593		
1	exm82031	10	variant.13877	14	exm1117596		
1	exm82037	10	rs1934956	14	exm1117599		
1	exm1692490	10	newrs144284469	14	exm1126639		
1	kgp6076404	10	exm844176	14	rs3742377		
1	kgp15658851	10	exm844180	14	rs943881		
1	rs10305754	10	exm844184	14	rs1951576		
1	kgp15264178	10	kgp21944904	14	rs12435918		
1	rs10305751	10	GA009074	14	rs2146238		
1	kgp10026466	10	rs6164	14	exm1126658		
1	kgp5045619	10	exm852644	14	exm1126669		
1	rs11552229	10	exm-rs17115100	15	rs2255192		
1	kgp15686930	10	rs17115100	15	rs4646		
1	kgp15816954	10	exm2216627	15	rs10046		
1	kgp15762369	10	exm852653	15	exm1161537		
1	exm95188	10	rs1042386	15	variant.35737		

Genetic variants were included in the analysis. CHR: chromosome, SNP: single nucleotide polymorphism.

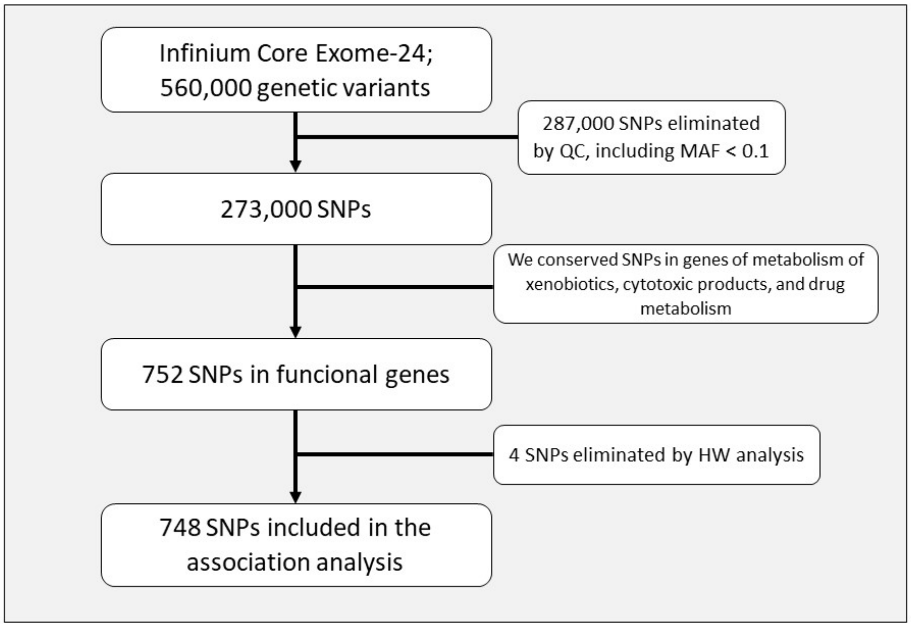

Supplementary Figure S3. SNPs filtering flowchart for SNPs association analysis.

Supplementary Table S2. Molecular data for SNPs associated in the smokers' comparison.

Gene	SNP	Chromosome	Change	Het. (O)	Het. (E)	p
CYP2J2	rs11572191	1	[G/A]	0.09	0.11	0.15
MGST3	rs8133	1	[G/T]	0.29	0.34	0.10
	rs4147611	1	[G/T]	0.46	0.49	0.34
ARNTL2	rs17497857	12	[T/C]	0.13	0.15	0.14
	rs4964059	12	[A/C]	0.17	0.19	0.27
MGST1	rs6488842	12	[C/T]	0.39	0.36	0.35
CYP46A1	rs3742377	14	[G/A]	0.37	0.33	0.22
	rs943881	14	[T/C]	0.34	0.33	0.53
	rs1951576	14	[A/G]	0.34	0.32	0.52
ARNT2	rs8041826	15	[G/A]	0.15	0.16	0.66
	rs3901896	15	[C/T]	0.48	0.49	0.89

Genes included in the table were associated in tobacco smoking comparison. Significant differences in the proportion of heterozygosity observed vs. expected were considered when $p < 0.05$. Het. (O): Observed heterozygosity, Het (E): Expected heterozygosity.

Supplementary Table S3. Molecular data for SNPs associated in exposed biomass-burning smoke comparison.

Gene	SNP	Chromosome	Change	Het. (O)	Het. (E)	p
MGST3	rs9333378	1	[G/A]	0.24	0.26	0.17
	rs11799886	1	[G/A]	0.04	0.04	1.00
CYP2J2	rs2229189	1	[G/A]	0.35	0.39	0.09
GSTM3	rs3814309	1	[T/C]	0.53	0.49	0.29
	rs4147611	1	[G/T]	0.31	0.36	0.05
	rs6681	1	[C/T]	0.01	0.01	1.00
	rs7483	1	[C/T]	0.52	0.49	0.41
	rs8133	1	[G/T]	0.16	0.16	1.00
	rs9333413	1	[G/A]	0.47	0.46	0.89
	rs957644	1	[C/T]	0.07	0.08	0.36
ADRB1	rs1801253	10	[G/A]	0.05	0.06	0.24

CYP2C8	rs10509681	10	[T/C]	0.03	0.04	0.09
	rs1934953	10	[C/T]	0.29	0.29	1.00
CYP2C9	rs1856908	10	[T/G]	0.25	0.26	0.49
	rs3752988	10	[T/C]	0.17	0.17	0.74
	rs9332220	10	[G/A]	0.09	0.09	0.49
GSTP1	rs1138272	11	[C/T]	0.01	0.01	1.00
CYP2R1	rs12794714	11	[C/A]	0.5	0.49	1.00
	rs7129781	11	[T/C]	0.03	0.03	1.00
ARNTL2	rs12297276	12	[G/A]	0.003	0.003	1.00
	rs4964059	12	[A/C]	0.15	0.15	0.44
	rs17497857	12	[T/C]	0.09	0.09	1.00
MGST1	rs1913263	12	[G/A]	0.51	0.48	0.33
	rs1042669	12	[T/G]	0.46	0.46	1.00
	rs2160512	12	[A/G]	0.33	0.31	0.25
	rs4149197	12	[G/C]	0.48	0.43	0.08
	rs9332959	12	[G/T]	0.45	0.45	1.00
ARNT2	rs3901896	15	[C/T]	0.36	0.36	1.00
	rs4238518	15	[G/A]	0.22	0.24	0.13
	rs1374213	15	[C/T]	0.25	0.29	0.3
	rs7168908	15	[A/G]	0.15	0.18	0.4
	rs7178902	15	[T/C]	0.20	0.21	0.58

Genes included in the table were associated in biomass burning exposure comparison. Significant differences in the proportion of heterozygosity observed vs. expected were considered when $p < 0.05$. Het. (O): Observed heterozygosity, Het (E): Expected heterozygosity.

Supplementary Table S4. Alleles data for smokers' comparison with no correction.

SNP/Alleles	COPD-S (n = 141)	AF %	SWOC (n = 213)	AF %	p
rs11572191					
C	245	86.88	401	94.13	1.00 (Ref.)
T	37	13.12	25	5.87	0.0008
rs8133					
G	197	69.86	335	78.64	1.00 (Ref.)
T	85	30.14	91	21.36	0.008
rs4147611					
G	163	57.80	205	48.12	1.00 (Ref.)
T	119	42.20	221	51.88	0.011
rs10741616					
G	140	49.65	246	57.75	1.00 (Ref.)
A	140	49.65	180	42.25	0.043
rs17497857					
T	246	87.23	388	91.08	1.00 (Ref.)
A	36	12.77	34	7.98	0.041
rs3742377					
G	240	85.11	336	78.87	1.00 (Ref.)
A	42	14.89	90	21.13	0.037
rs1037124					
G	240	85.11	389	91.31	1.00 (Ref.)
A	42	14.89	37	8.69	0.01
exm1161562					
G	272	96.45	393	92.25	1.00 (Ref.)
A	10	3.55	33	7.75	0.022
rs3901896					
T	137	48.58	243	57.04	1.00 (Ref.)
C	145	51.42	183	42.96	0.027
rs16964220					
G	263	93.26	376	88.26	1.00 (Ref.)
A	19	6.74	50	11.74	0.03
rs10519301					
G	273	96.81	422	99.06	1.00 (Ref.)
A	9	3.19	4	0.94	0.029
exm-rs16964211					
G	262	92.91	375	88.03	1.00 (Ref.)
A	20	7.09	51	11.97	0.034

Comparison of allele frequencies by χ^2 We considered significant association when $p < 0.05$. AF: Allele frequency %, SNP: Single Nucleotide Polymorphism, Ref: Reference.

Supplementary Table S5. Genotype data for smokers' comparison with no correction.

SNP/Alleles	COPD-S (n = 141)	GF %	SWOC (n = 213)	GF %	p
rs943881					
TT	85	60.28	133	62.44	1.00 (Ref.)
TC	41	29.08	73	34.27	0.017
CC	15	10.64	7	3.29	
rs1951576					
AA	85	60.28	133	62.44	1.00 (Ref.)
AG	41	29.08	73	34.27	0.017
GG	15	10.64	7	3.29	
rs8133					
GG	70	49.65	136	63.85	1.00 (Ref.)
GT	57	40.43	63	29.58	0.029
TT	14	9.93	14	6.57	
rs6488842					
CC	79	56.03	122	57.28	1.00 (Ref.)
CT	46	32.62	82	38.50	0.03
TT	16	11.35	9	4.23	

Comparison of allele frequencies by χ^2 We considered significant association when $p < 0.05$. GF: Genotype frequency, %, SNP: Single Nucleotide Polymorphism, Ref: Reference.

Supplementary Table S6. Alleles data in exposed biomass-burning smoke comparison with no correction.

SNP/Alleles	COPD-BBS (n = 98)	AF %	BBES (n = 293)	AF %	OR	CI (95%)	p
rs4147611							
T	125	63.78	450	76.79		1.00 (Ref.)	
G	71	36.22	136	23.21	1.94	1.33 - 2.85	0.0007
rs11799886							
G	16	8.16	12	2.05		1.00 (Ref.)	
A	180	91.84	574	97.95	3.96	1.66 - 9.49	0.002
exm119609							
C	190	96.94	582	99.32		1.00 (Ref.)	
T	6	3.06	4	0.68	8.99	1.85 - 43.78	0.007
newrs9333378							
A	149	76.02	497	84.81		1.00 (Ref.)	
G	47	23.98	89	15.19	1.72	1.12 - 2.64	0.013
rs957644							
C	176	89.80	563	96.08		1.00 (Ref.)	
T	20	10.20	23	3.92	2.26	1.17 - 4.37	0.015
exm57446							
C	90	45.92	308	52.56		1.00 (Ref.)	
T	106	54.08	278	47.44	1.58	1.07 - 2.34	0.021
rs10789501							
C	90	45.92	308	52.56		1.00 (Ref.)	
T	106	54.08	278	47.44	1.58	1.07 - 2.34	0.021
rs6690005							
A	92	46.94	306	52.22		1.00 (Ref.)	
G	104	53.06	280	47.78	1.55	1.06 - 2.29	0.026
kgp8583761							
T	186	94.90	577	98.46		1.00 (Ref.)	
C	10	5.10	9	1.54	15.06	1.38 - 164	0.026
rs1856908							
T	139	70.92	498	84.98		1.00 (Ref.)	
G	57	29.08	88	15.02	2.05	1.31 - 3.19	0.002
rs1934953							
G	135	68.88	482	82.25		1.00 (Ref.)	
A	61	31.12	104	17.75	2.01	1.29 - 3.12	0.002
rs3752988							
T	160	81.63	530	90.44		1.00 (Ref.)	
C	36	18.37	56	9.56	2.06	1.19 - 3.57	0.01
rs9332220							

G	173	88.27	558	95.22		1.00 (Ref.)	
A	23	11.73	28	4.78	2.29	1.15 - 4.56	0.019
exm857200							
C	179	91.33	568	96.93		1.00 (Ref.)	
G	17	8.67	18	3.07	2.49	1.13 - 5.53	0.024
exm844097							
T	184	93.88	575	98.12		1.00 (Ref.)	
C	12	6.12	11	1.88	2.73	1.09 - 6.86	0.033
rs12794714							
G	118	60.20	298	50.85		1.00 (Ref.)	
A	78	39.80	288	49.15	0.54	0.36 - 0.81	0.0026
rs1138272							
C	192	97.96	583	99.49		1.00 (Ref.)	
T	4	2.04	3	0.51	8.95	1.563 - 51.22	0.014
rs7129781							
T	186	94.90	576	98.29		1.00 (Ref.)	
C	10	5.10	10	1.71	2.97	1.039 - 8.49	0.042
rs1913263							
G	90	45.92	350	59.73		1.00 (Ref.)	
A	106	54.08	236	40.27	1.86	1.26 - 2.735	0.002
kgp18860019							
T	147	75.00	380	64.85		1.00 (Ref.)	
G	49	25.00	206	35.15	0.61	0.39 - 0.94	0.024
rs9332959							
G	147	75.00	381	65.02		1.00 (Ref.)	
T	49	25.00	205	34.98	0.63	0.41 - 0.96	0.031
rs4149197							
G	115	58.67	399	68.09		1.00 (Ref.)	
C	81	41.33	187	31.91	1.52	1.01 - 2.28	0.044
rs11048977							
G	151	77.04	409	69.80		1.00 (Ref.)	
A	45	22.96	177	30.20	0.64	0.48 - 0.99	0.047
exm-rs2899472							
C	187	95.41	573	97.78		1.00 (Ref.)	
A	9	4.59	13	2.22	2.9	1.09 - 7.72	0.033
kgp3956993							
G	193	98.47	585	99.83		1.00 (Ref.)	
A	3	1.53	1	0.17	11.67	1.08 - 126.5	0.043

Comparison of allele frequencies by χ^2 We considered significant association when $p < 0.05$. AF: Allele frequency, %, SNP: Single Nucleotide Polymorphism, Ref: Reference.

Supplementary Table S7. Genotype data in exposed biomass-burning smoke comparison with no correction.

SNP/Alleles	COPD-BBS (n = 98)	GF %	BBES (n = 293)	GF %	OR	CI (95%)	p
kgp8583761							
TT	88	89.80	284	96.93		1.00 (Ref.)	
TG	10	10.20	9	3.07	15.44	1.79 - 335.6	0.025
GG	0	0	0	0.00	NA	NA	NA
rs6690005							
AA	21	21.43	81	27.65		1.00 (Ref.)	
AG	50	51.02	144	49.15	1.65	0.81 - 3.45	0.17
GG	27	27.55	68	23.21	2.86	1.26 - 6.69	0.013
exm57446							
CC	19	19.39	82	27.99		1.00 (Ref.)	
CT	52	53.06	144	49.15	1.75	0.87 - 3.68	0.13
TT	27	27.55	67	22.87	2.84	1.24 - 6.73	0.015
newrs9333378							
AA	59	60.20	214	73.04		1.00 (Ref.)	
AG	31	31.63	69	23.55	1.89	1.02 - 3.49	0.041
GG	8	8.16	10	3.41	2.79	8.32 - 9.29	0.092
rs9333413							
AA	39	39.80	120	40.96		1.00 (Ref.)	
AG	36	36.73	136	46.42	0.91	0.48 - 1.72	0.77
GG	23	23.47	36	12.29	2.22	1.03 - 4.79	0.042
rs957644							
CC	80	81.63	271	92.49		1.00 (Ref.)	
CT	16	16.33	21	7.17	2.29	1.02 - 5.07	0.041
TT	2	2.04	1	0.34	5.26	0.48 - 116.3	0.18
exm119609							
CC	92	93.88	289	98.63		1.00 (Ref.)	
CT	6	6.12	4	1.37	9.77	2.11 - 54.79	0.005
TT	0	0.00	0	0.00	NA	NA	NA
rs11799886							
GG	82	83.67	281	95.90		1.00 (Ref.)	
GA	16	16.33	12	4.10	4.51	1.82 - 11.47	0.001
AA	0	0.00	0	0.00	NA	NA	NA
rs8133							
GG	74	75.51	243	82.94		1.00 (Ref.)	
GT	19	19.39	48	16.38	1.32	0.63 - 2.67	0.45
TT	5	5.10	2	0.68	12.44	2.31 - 99.26	0.006
rs4147611							

TT	43	43.88	179	61.09		1.00 (Ref.)	
TG	39	39.80	92	31.40	2.04	1.09 - 3.80	0.025
GG	16	16.33	22	7.51	4.57	1.88 - 11.21	0.0008
rs1856908							
TT	50	51.02	213	72.70		1.00 (Ref.)	
TG	39	39.80	72	24.57	2.51	1.36 - 4.67	0.003
GG	9	9.18	8	2.73	4.59	1.43 - 14.88	0.009
rs9332220							
GG	77	78.57	266	90.78		1.00 (Ref.)	
GA	19	19.39	26	8.87	2.41	1.07 - 5.33	0.031
AA	2	2.04	1	0.34	2.57	0.09 - 66.83	0.51
rs1934953							
GG	47	47.96	198	67.58		1.00 (Ref.)	
GA	41	41.84	86	29.35	2.53	1.38 - 4.68	0.0027
AA	10	10.20	9	3.07	4.93	1.54 - 16.18	0.0072
exm844097							
TT	87	88.78	283	96.59		1.00 (Ref.)	
TC	10	10.20	9	3.07	4.69	1.48 - 14.87	0.007
CC	1	1.02	1	0.34	3.13	0.12 - 84.55	0.44
rs3752988							
TT	64	65.31	240	81.91		1.00 (Ref.)	
TC	32	32.65	50	17.06	2.98	1.54 - 5.78	0.001
CC	2	2.04	3	1.02	4.5	0.45 - 45.74	0.18
exm857200							
CC	81	82.65	276	94.20		1.00 (Ref.)	
CG	17	17.35	16	5.46	3.01	1.24 - 7.24	0.014
GG	0	0.00	1	0.34	NA	NA	NA
rs12794714							
GG	36	36.73	76	25.94		1.00 (Ref.)	
GA	46	46.94	146	49.83	0.47	0.25 - 0.89	0.022
AA	16	16.33	71	24.23	0.35	0.14 - 0.79	0.015
rs1913263							
GG	22	22.45	100	34.13		1.00 (Ref.)	
GA	46	46.94	150	51.19	1.41	0.71 - 2.91	0.33
AA	30	30.61	43	14.68	3.42	1.57 - 7.68	0.002
rs4149197							
GG	35	35.71	129	44.03		1.00 (Ref.)	
GC	45	45.92	141	48.12	0.96	0.51 - 1.79	0.89
CC	18	18.37	23	7.85	3.73	1.56 - 9.06	0.003
kgp18860019							
TT	56	57.14	123	41.98		1.00 (Ref.)	

TG	35	35.71	134	45.73	0.44	0.24 - 0.79	0.007
GG	7	7.14	36	12.29	0.5	0.17 - 1.32	0.18
rs9332959							
GG	56	57.14	124	42.32		1.00 (Ref.)	
GT	35	35.71	133	45.39	0.46	0.25 - 0.83	0.01
TT	7	7.14	36	12.29	0.52	0.17 - 1.34	0.19
exm-rs2899472							
CC	89	90.82	280	95.56		1.00 (Ref.)	
CA	9	9.18	13	4.44	3.2	1.17 - 8.57	0.021
AA	0	0.00	0	0.00	NA	NA	NA
kgp3956993							
GG	95	96.94	292	99.66		1.00 (Ref.)	
GA	3	3.06	1	0.34	12.9	1.45 - 2.8	0.036
AA	0	0.00	0	0	NA	NA	NA

Comparison of allele frequencies by χ^2 We considered significant association when $p < 0.05$. GF: Genotype frequency, %, SNP: Single Nucleotide Polymorphism, Ref: Reference. NA: Not applicable.

Supplementary Table S8. Analysis by severity in patients with COPD-S.

SNP/Alleles	Severe states (n = 74)	Mild states (n = 88)	OR	CI (95%)	p*	p§	p~
rs12435918	*CYP46A1*						
A	48.89	67.19					
G	51.11	32.81	2.14	1.28-3.57	0.003	0.007	NS
rs625456	*GSTM2*						
G	91.11	97.4					
T	8.89	2.6	3.65	1.16-11.49	0.019	0.035	NS
rs11509438	*GSTO1*						
G	95.56	99.48					
A	4.44	0.52	8.88	0.98-80.66	0.019	NS	NS
rs2146238	*CYP46A1*						
G	86.67	75.0					
T	13.33	25.0	0.46	0.23-0.92	0.026	0.03	NS
rs1801253	*ADRB1*						
C	80.0	89.47					
G	20.0	10.53	2.13	1.06-4.25	0.031	NS	NS
rs4238518	*ARNT2*						
A	90.0	80.73					
G	10.0	19.27	0.47	0.21-1.01	0.049	NS	NS
rs1058930	*CYP2C8*						
G	94.44	97.92					
C	5.56	2.08	2.76	0.7-10.55	NS	0.043	NS

Association analysis stratifying alleles frequency by the severity of COPD-S. Frequencies were compared by Fisher exact test without correction (*), covariates correction (§), and Bonferroni adjustment (~). A significant association was considered when $p < 0.05$. Severe stages: GOLD 3 + GOLD 4, Mild states: GOLD 1 + GOLD 2, OR: Odds ratio, CI: Confidence interval, NS: No significant.

Supplementary Table S9. Analysis by severity in patients with COPD-BBES.

SNP/Alleles	Sever states (n = 12)	Mild states (n = 75)	OR	CI (95%)	p*	p§	p~
rs12300289	*ARNTL2*						
A	88.46	99.42					
T	11.54	0.58	22.04	2.2-220.9	0.0002	0.009	NS
rs10847	*ARNT*						
C	57.69	81.55					
T	42.31	18.45	3.24	1.36-7.74	0.006	0.013	NS
rs2234696	*GSTM3*						
T	88.46	98.23					
G	11.54	1.77	7.26	1.38-38.13	0.007	0.023	NS
rs4778795	*ARNT2*						
G	80.77	58.93					
T	19.23	41.07	0.34	0.12-0.95	0.032	0.038	NS
rs1562048	*ARNTL2*						
G	73.08	88.24					
A	26.92	11.76	2.76	1.03-7.39	0.037	NS	NS
rs2036462	*CYP4X1*						
T	76.92	60.59					
C	23.08	39.41	0.46	0.17-1.21	NS	0.039	NS
rs11211422	*CYP4X1*						
G	73.08	58.93					
A	26.92	41.07	0.53	0.21-1.32	NS	0.049	NS

Association analysis stratifying alleles frequency by the severity of COPD-BBS. Frequencies were compared by Fisher exact test without correction (*), covariates correction (§), and Bonferroni adjustment (~). A significant association was considered when $p < 0.05$. Severe stages: GOLD 3 + GOLD 4, Mild states: GOLD 1 + GOLD 2, OR: Odds ratio, CI: Confidence interval, NS: No significant.

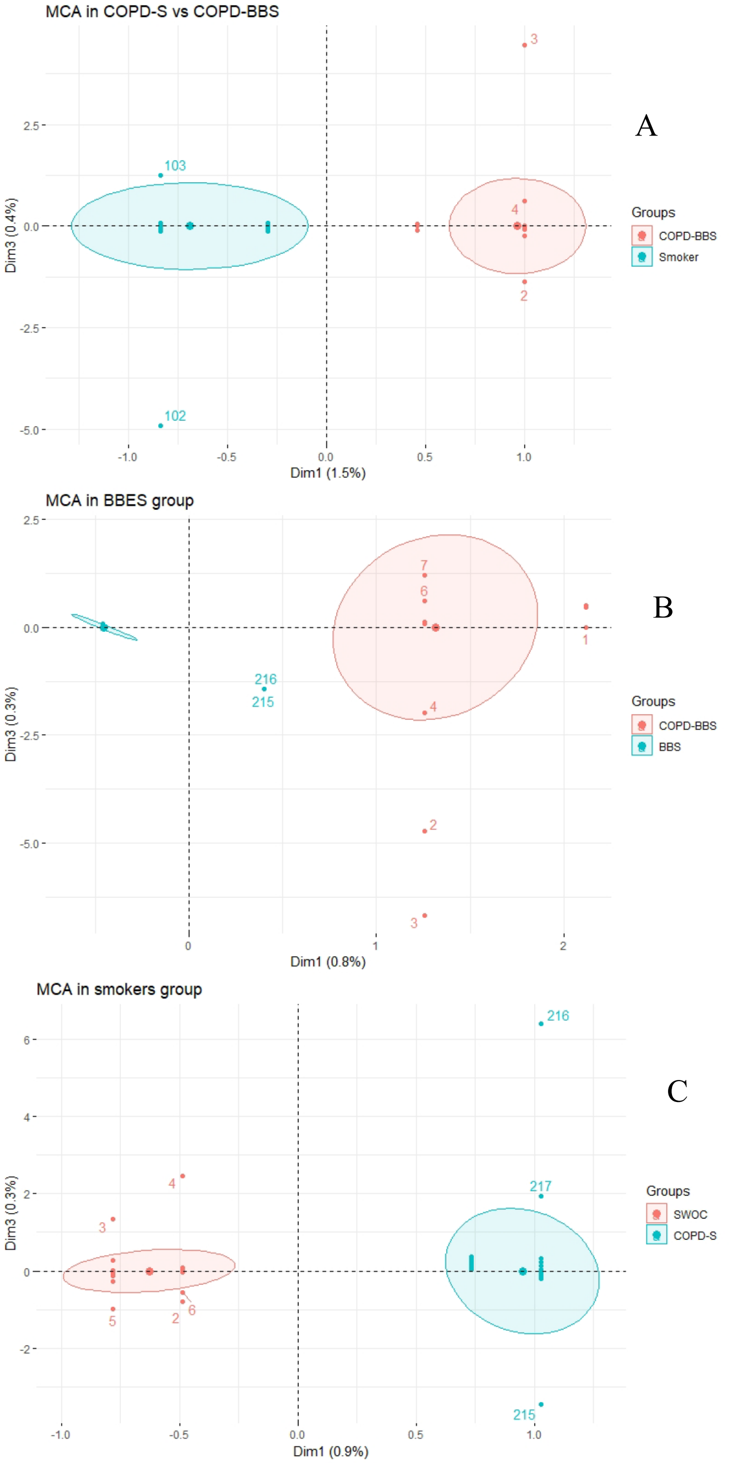

Supplementary Figure S4. Multiple Correspondence Analysis (MCA), including all SNPs with MAF > 1%. A) MCA for COPD stratified by environmental risk. B) Analysis including smokers' comparison. C) MCA for BBES comparison.

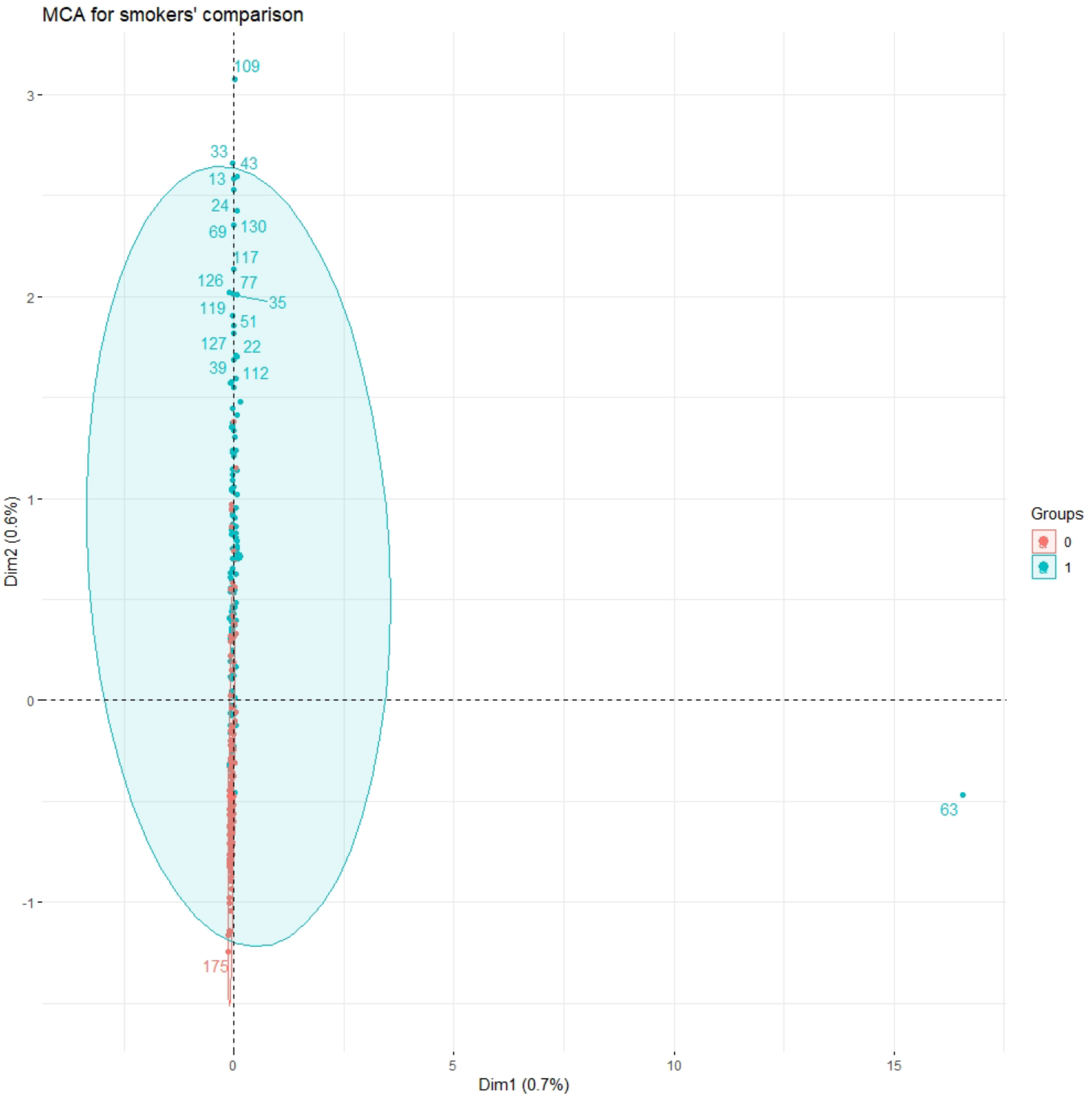

Supplementary Figure S5. Multiple correspondence analysis (MCA) for smokers' comparison. We did not find a possible grouping between the SNPs and quantitative variables. 0 = SWOC, 1 = COPD-S.

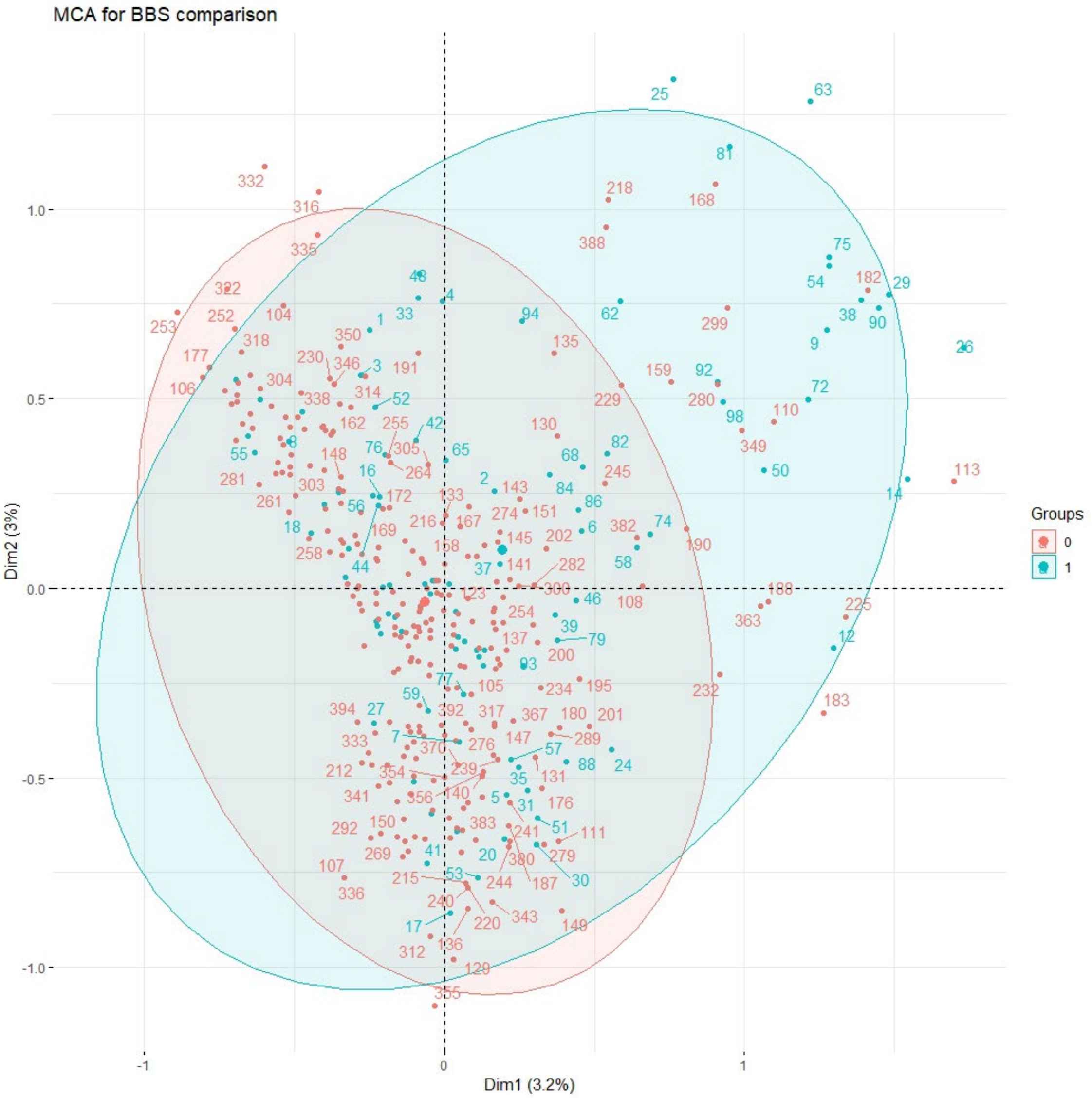

Supplementary Figure S6. Multiple correspondence analysis (MCA) for biomass' comparison. We did not find a possible grouping between the SNPs and quantitative variables. 0 = BBES, 1 = COPD-BBS.

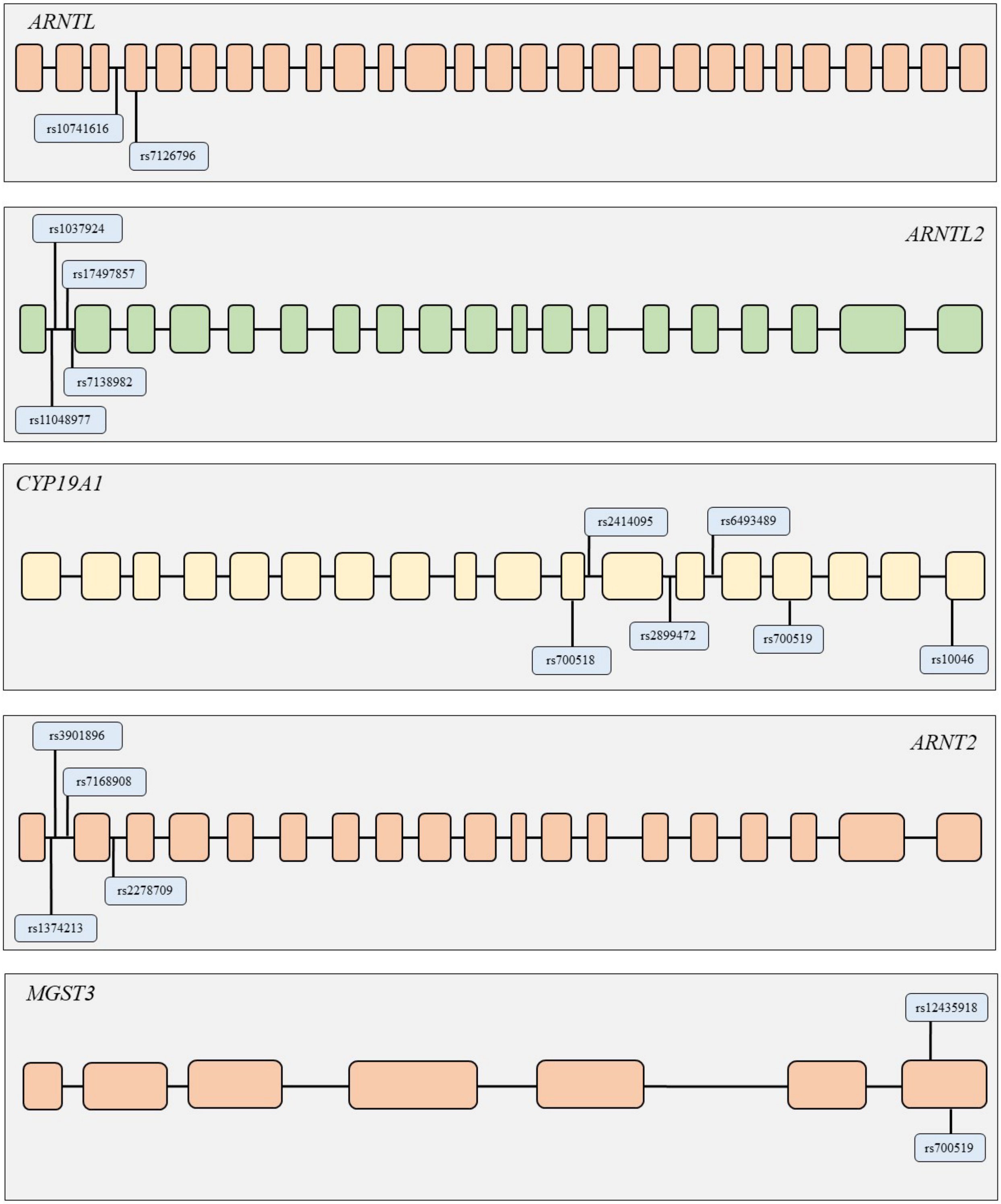
ARNTL
rs10741616
rs7126796
rs1037924
ARNTL2
rs17497857
rs7138982
rs11048977
CYP19A1
rs2414095
rs6493489
rs700518
rs2899472
rs700519
rs10046
rs3901896
ARNT2
rs7168908
rs2278709
rs1374213
MGST3
rs12435918
rs700519

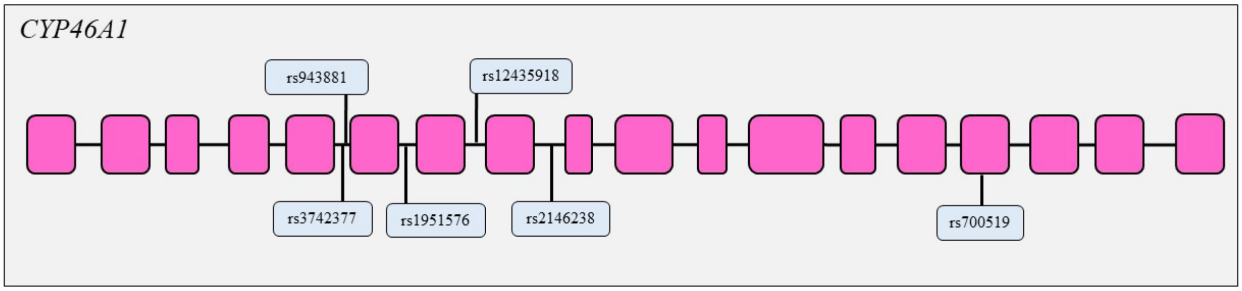

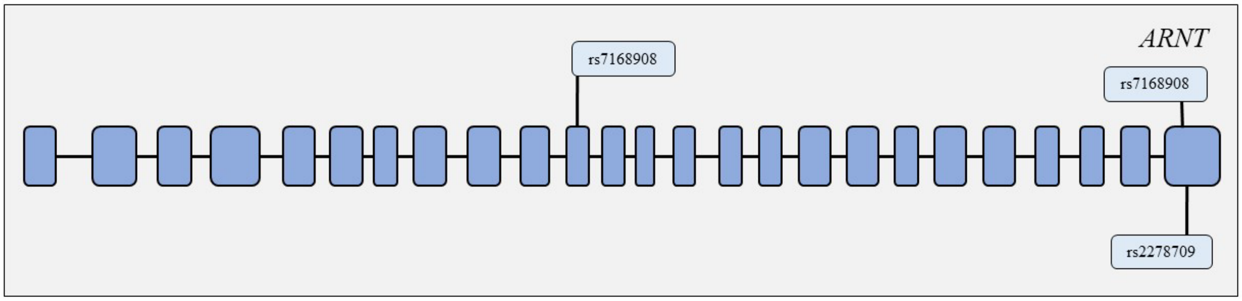

Supplementary Figure S7. Gene schemes with the SNPs associated in haplotype analysis in smokers' comparison.

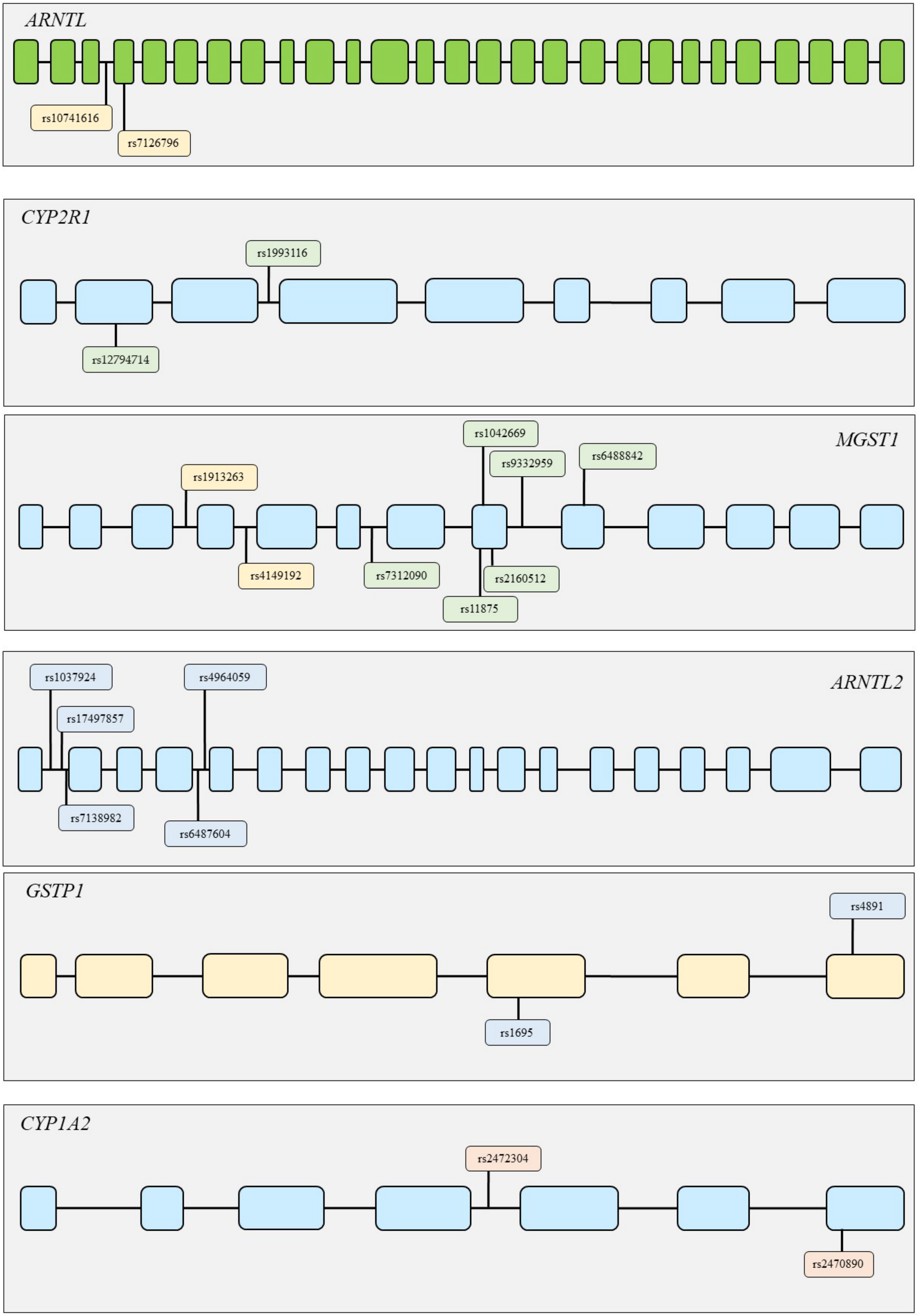
ARNTL
rs10741616
rs7126796
CYP2R1
rs1993116
rs12794714
MGST1
rs1042669
rs9332959
rs6488842
rs1913263
rs4149192
rs7312090
rs2160512
rs11875
ARNTL2
rs1037924
rs4964059
rs17497857
rs7138982
rs6487604
GSTP1
rs4891
rs1695
CYP1A2
rs2472304
rs2470890

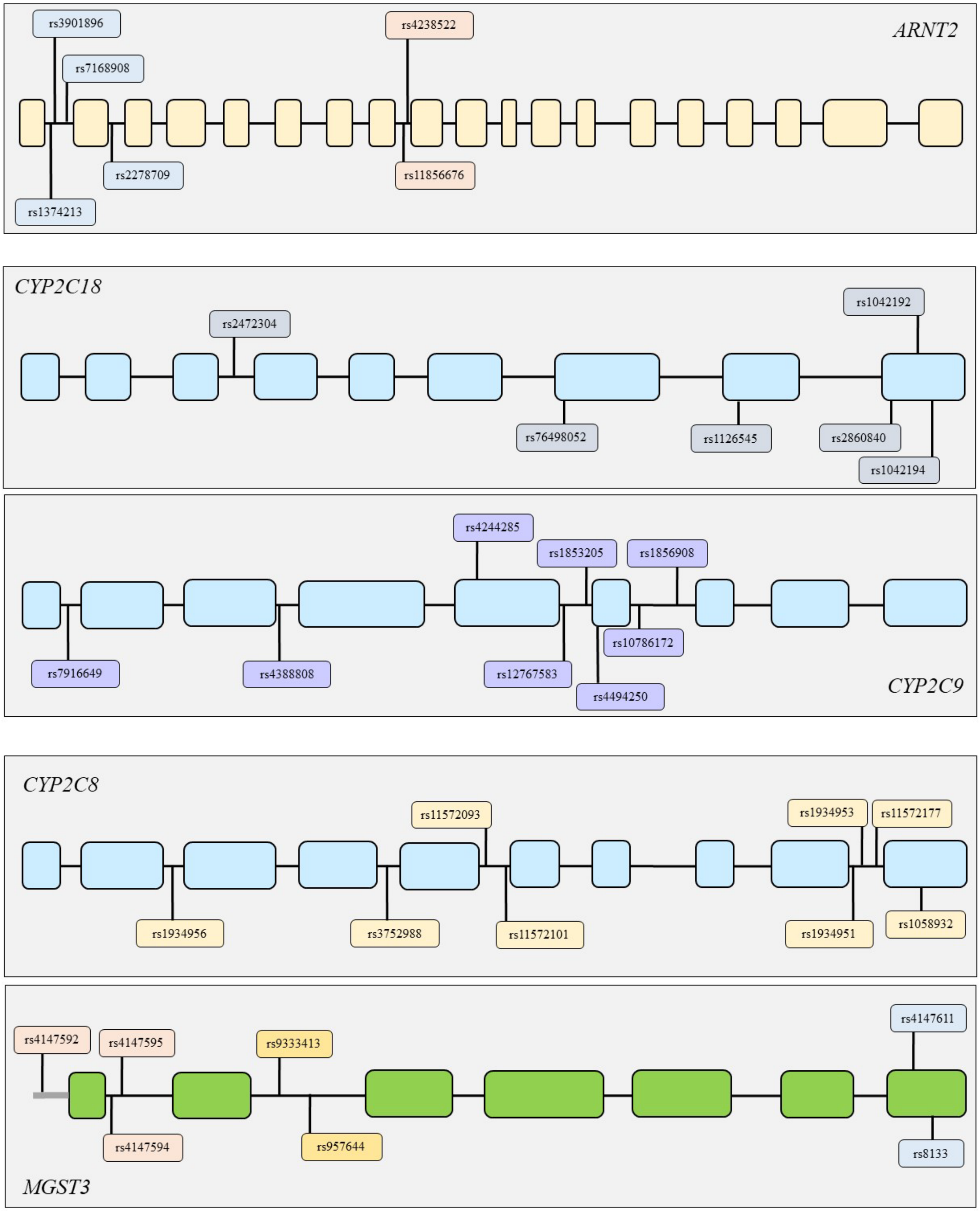
ARNT2
rs3901896
rs7168908
rs4238522
rs2278709
rs11856676
rs1374213
CYP2C18
rs2472304
rs1042192
rs76498052
rs1126545
rs2860840
rs1042194
rs4244285
rs1853205
rs1856908
rs10786172
rs7916649
rs4388808
rs12767583
rs4494250
CYP2C9
CYP2C8
rs11572093
rs1934953
rs11572177
rs1934956
rs3752988
rs11572101
rs1934951
rs1058932
rs4147592
rs4147595
rs9333413
rs4147611
rs4147594
rs957644
rs8133
MGST3

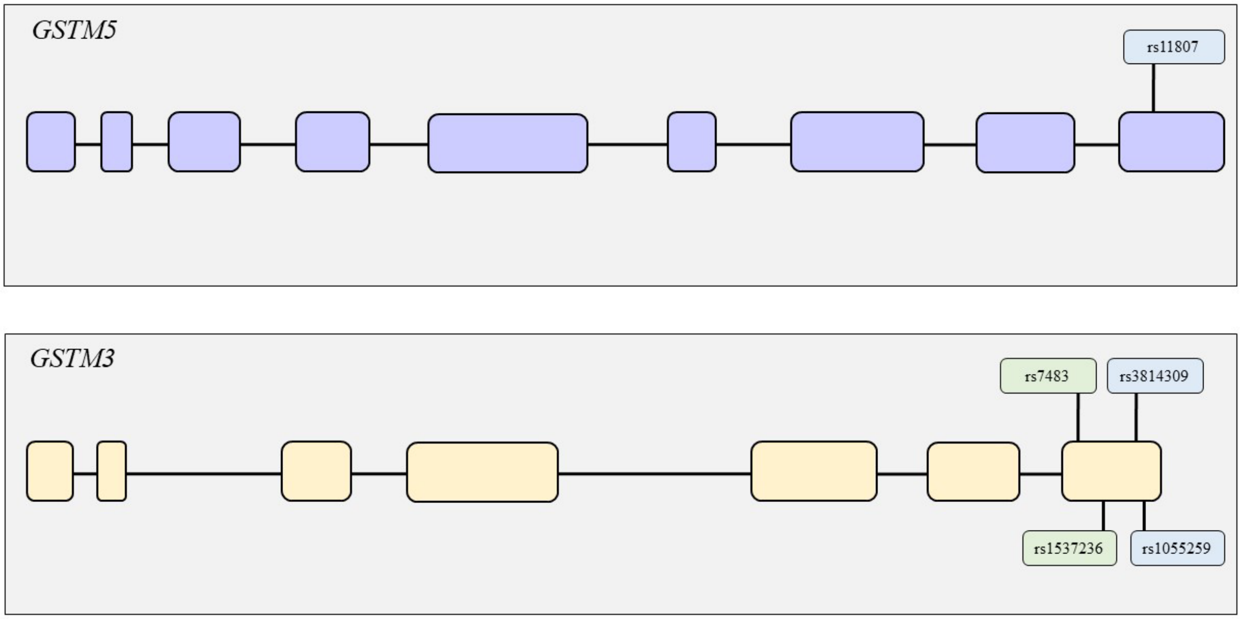

Supplementary Figure S8. Gene schemes with the SNPs associated with haplotype analysis in biomass comparison.
